# Enhancing educational and vocational recovery in adolescents and young adults with early psychosis through Supported Employment and Education (SEEearly): study protocol for a multicenter randomized controlled trial

**DOI:** 10.1186/s13063-023-07462-2

**Published:** 2023-07-03

**Authors:** D. Jäckel, A. Willert, A. Brose, K. Leopold, D. Nischk, S. Senner, O. Pogarell, S. Sachenbacher, M. Lambert, A. Rohenkohl, P. Kling-Lourenco, N. Rüsch, F. Bermpohl, M. Schouler-Ocak, V. Disselhoff, U. Skorupa, A. Bechdolf

**Affiliations:** 1 Department of Psychiatry and Psychotherapy, Charité Campus Mitte, Charité Universitätsmedizin Berlin, Berlin, Germany; 2 Department of Psychiatry, Psychotherapy and Psychosomatics, Vivantes Klinikum am Urban and Vivantes Klinikum im Friedrichshain, Berlin, Germany; 3 Department of Psychiatry and Psychotherapy, University Hospital Carl Gustav Carus, Technical University Dresden, Dresden, Germany; 4 Department of Social Psychiatry, Zentrum für Psychiatrie, Reichenau, Germany; 5 Department of Psychiatry and Psychotherapy, University Hospital, LMU Munich, Munich, Germany; 6 Department of Psychiatry and Psychotherapy, University Medical Center Hamburg-Eppendorf, Hamburg, Germany; 7 Department of Psychiatry II, University of Ulm and BKH Günzburg, Ulm, Germany; 8 Psychiatric University Clinic of Charité at St. Hedwig Hospital, Berlin, Germany

**Keywords:** IPS, Early psychosis, Schizophrenia, Early intervention, Recovery

## Abstract

**Background:**

Psychotic disorders often develop a chronic course with devastating consequences for individuals, families, and societies. Early intervention programs for people in the first 5 years after the initial psychotic episode (early psychosis) can significantly improve the outcome and are therefore strongly recommended in national and international guidelines. However, most early intervention programs still focus on improving symptoms and relapse prevention, rather than targeting educational and vocational recovery. The aim of the present study is to explore the effects of Supported Employment and Education (SEE) following the Individual Placement and Support (IPS) model in people with early psychosis.

**Methods:**

The SEEearly trial compares treatment as usual (TAU) plus SEE to TAU alone in outpatient psychiatric settings. The study is a six-site, two-arm, single-blinded, superiority randomized controlled trial (RCT). Participants are randomly assigned (1:1) to the intervention or control group. Aiming to recruit 184 participants, with an assumed drop-out rate of 22%, we will be able to detect a 24% difference in the main outcome of employment/education with 90% power. We make assessments at baseline and at 6- and 12-month follow-ups. Outcome data on employment/education, medication, and current psychiatric treatment is obtained monthly through phone based short assessments. The primary outcome is steady participation for at least 50% of the 12-month follow-up in competitive employment and/or mainstream education. Secondary employment outcomes capture length of employment/education, time to first employment/education, monthly wages/educational attainment, and social return on investment (SROI). Secondary non-employment outcomes include subjective quality of life, psychopathology, substance use, relapse, hospitalization, and functional impairment. To be eligible, participants must be between 16 and 35 years, fulfill diagnostic criteria for early psychosis, and be interested in competitive employment and/or mainstream education.

**Discussion:**

In SEEearly, we hypothesize that participants with psychosis, who receive TAU plus SEE, present with better primary and secondary outcomes than participants, who receive TAU alone. Positive results of this study will justify SEE as an evidence-based strategy for clinical routine treatment in people with early psychosis.

**Trial registration:**

SEEearly was registered nationally and internationally in the German Clinical Trials Register (DRKS; identifier: DRKS00029660) on October 14, 2022.

## Administrative information

Note: the numbers in curly brackets in this protocol refer to SPIRIT checklist item numbers. The order of the items has been modified to group similar items (see http://www.equator-network.org/reporting-guidelines/spirit-2013-statement-defining-standard-protocol-items-for-clinical-trials/).

**Table:** 

Title {1}	The SEEearly trial is a six site, two-arm, single blinded RCT exploring the effects of Supported Employment and Education (SEE) following the Individual Placement and Support (IPS) model in people with early psychosis.
Trial registration {2a} and {2b}.	German Clinical Trials Register (DRKS); identifier: DRKS00029660; registered October 14^th^, 2022.
Protocol version {3}	This version refers to version 1.2 of the approved protocol (September 15, 2022).
Funding {4}	The project is funded by third-party-funds by the German Research Foundation (DFG) over a period of 3 years.
Author details {5a}	D Jäckel*^1,2^, A Willert*^1,2^, A Brose^2^, K Leopold^2,8^, D Nischk^3^, S Senner^3^, O Pogarell^4^, S Sachenbacher^4^, M Lambert^5^, A Rohenkohl^5^, P Kling-Lourenco^6^, N Rüsch^6^, F Bermpohl^1,7^, M Schouler-Ocak^1,7^, V Disselhoff^2^, U Skorupa^2^, A Bechdolf^1,2^ *shared first authorship ^1^ Department of Psychiatry and Psychotherapy, Charité Campus Mitte, Charité Universitätsmedizin Berlin, Berlin, Germany ^2^ Department of Psychiatry, Psychotherapy and Psychosomatics, Vivantes Klinikum am Urban and Vivantes Klinikum im Friedrichshain, Berlin, Germany ^3^ Department of Social Psychiatry, Zentrum für Psychiatrie, Reichenau, Germany ^4^ Department of Psychiatry and Psychotherapy, University Hospital, LMU Munich, Munich Germany ^5^ Department of Psychiatry and Psychotherapy, University Medical Center Hamburg-Eppendorf, Hamburg, Germany ^6^ Department of Psychiatry II, University of Ulm and BKH Günzburg, Ulm, Germany ^7^ Psychiatric University Clinic of Charité at St. Hedwig Hospital, Berlin, Germany ^8^ Department of Psychiatry and Psychotherapy, University Hospital Carl Gustav Carus, Technical University Dresden, Dresden, Germany
Name and contact information for the trial sponsor {5b}	Department of Psychiatry and Psychotherapy, Charité Campus Mitte, Charité Universitätsmedizin Berlin, Charitéplatz 1, 10117 Berlin, Germany
Role of sponsor {5c}	The Department of Psychiatry and Psychotherapy, Charité Campus Mitte functions as the study sponsor and receives third-party-funds by the DFG. A Bechdolf as principal investigator, D Jäckel as scientific project coordinator and A Willert as transregional project coordinator are partially funded by the DFG.

## Introduction

### Background and rationale {6a}

Psychotic disorders often lead to a chronic course with devastating consequences for individuals, families, and societies, usually with first onset during adolescence or early adulthood [1, 2] when individuals are typically pursuing their education, employment, and career trajectories. The low rates of completing secondary education and obtaining competitive employment with education, additionally to vocational recovery rates of only 13.5% [3] represent one of the main burdens in individuals and their families and account for the majority of costs the illness causes for societies [4, 5].

Early intervention programs, which provide intensive, phase specific, psychosocial, and pharmacological treatment for people in the first 5 years after the initial psychotic episode (a phase referred to as *early psychosis* [6]), can significantly improve the outcome and reduce negative consequences of early psychosis [7–9]. Because of these findings, national and international guidelines support the implementation of early intervention programs with a high level of recommendation [10–12]. However, to date, most early intervention programs for people with early psychosis still focus on improving symptoms and preventing relapse, rather than targeting educational and vocational recovery [7, 8, 13]. This is inappropriate because engagement in work and education has a high priority for young people with early psychosis and significantly reduces the social disability associated with the disorder [14, 15].

In the present trial, we investigate the effects of Supported Employment (SE) following the IPS model, which focuses on obtaining competitive work through improvement of functioning and vocational recovery in people with early psychosis [16, 17]. Furthermore, since many adolescents or young adults with early psychosis are still in secondary education, the IPS model is extended to achieve mainstream education: Supported Employment and Education – SEE [18]. There is strong evidence, that SE in adults with severe mental illness (SMI) leads to much higher rates of competitive job acquisition, increased working hours per week, and higher wages compared to general rehabilitation services [19–21]. Secondary education, SE, and competitive employment correlate positively with clinical, social, and economical outcomes as well as with quality of life [22–24]. Competitive employment is defined as jobs that anyone can apply for regardless of disability status. Mainstream education is defined in accordance with the Organization for Economic Cooperation and Development (OECD) [25] as educational programs leading to a qualifying degree and open to the general public. In Germany, this includes the following educational settings: secondary education (“sekundärer Bildungsbereich”), education examinations, internships (e.g., “Praktisches Jahr”), apprenticeship that generally lasts for 2 to 3 years and enable people to carry out a professional activity (“Berufsausbildung”), and higher education (university, university of applied sciences). Vocational education programs in line with traditional vocational rehabilitation (TVR) approaches following the “first train – then place” approach do not account as mainstream education [18].

Despite the proven effectiveness and recommendation in national and international guidelines [11, 12], there are only six RCTs of SEE in early psychosis available up to now [16, 20, 21, 26–28]. These include mostly young adults above 18 years, mainly focus on employment and not on both employment and education, sample sizes are limited in several of them, and none of the studies is a multi-site study. SEEearly therefore is the first multisite RCT of SEE in adolescents and young adults with early psychosis worldwide. SEEearly overcomes the limitations of the RCTs mentioned above. In particular, this involves (a) applying SEE rather than SE and (b) considering the primary outcome “steady participation for at least 50% of the 12-month follow-up period in competitive employment and/or mainstream education” rather than “at least one day”, (c) a sample size that is large enough to determine the relative effects of TAU plus SEE in comparison to TAU, (d) applying a meaningful intervention and follow-up time period, (e) using a standardized manual of SEE, (f) measuring the fidelity of the intervention by applying the IPS Fidelity Scale for Young Adults [18], and (g) applying an economical evaluation.

### Objectives {7}

The central hypothesis of SEEearly is that participants with early psychosis who receive TAU plus SEE show better competitive employment and/or mainstream education outcomes than participants who receive TAU alone. This article describes the protocol for the SEEearly trial.

### Trial design {8}

The SEEearly study is a six-site, prospective, rater blinded, two-arm, superiority RCT. After being assessed for eligibility, fulfilling inclusion but not exclusion criteria, and providing written informed consent, participants are randomized with a 1:1 ratio to either TAU plus SEE or to TAU alone.

**Fig. 1:**
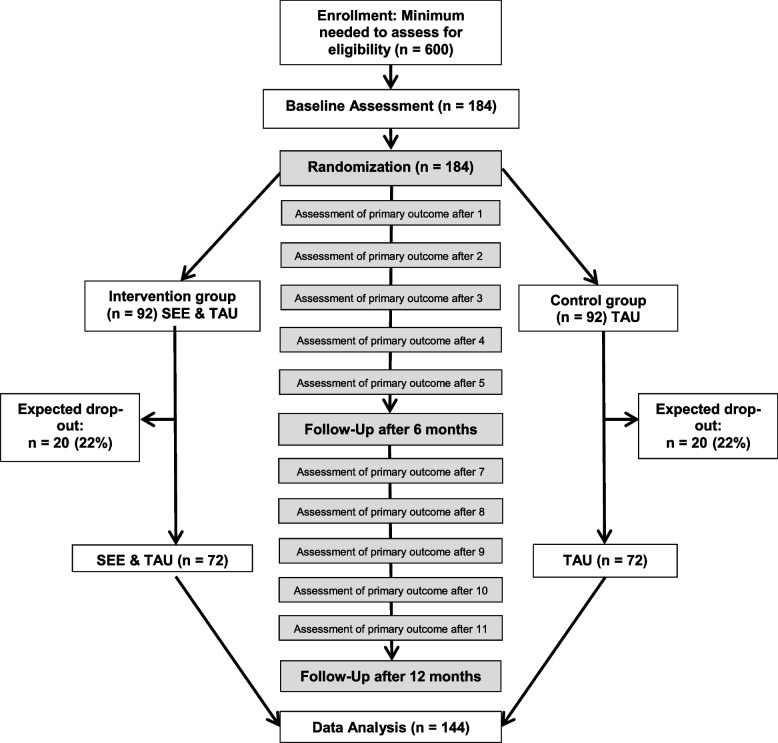
Trial timeline and design

The primary outcome and secondary employment outcomes, current medication, and current psychiatric treatment are assessed monthly. Assessments regarding further secondary outcomes are made at baseline and at 6 and 12 months of follow-up (see Fig. 1 and Table 1).

**Table 1:** Content and timeline of participant ratings

**Content of ratings**	**Baseline/t0**						**t1**						**t2**
**/month**	**Baseline**	**1**	**2**	**3**	**4**	**5**	**6**	**7**	**8**	**9**	**10**	**11**	**12**
**By rater**													
Diagnosis acc. to DSM-5 and ICD-10, sociodemographic background, living situation, expectations reg. employment/education	x												
Prior psychiatric treatment	x												
Ongoing psychiatric treatment	x	x	x	x	x	x	x	x	x	x	x	x	x
Ongoing medication	x	x	x	x	x	x	x	x	x	x	x	x	x
Primary outcome		x	x	x	x	x	x	x	x	x	x	x	x
Secondary outcome (employment and education)		x	x	x	x	x	x	x	x	x	x	x	x
Psychopathology (PANSS)	x						x						x
Functional impairment (Mini ICF-APP, GAF)	x						x						x
Social support	x						x						x
Substance use (ASI, DFAQ-CU)	x						x						x
Social return on investment													x
**Self-rating**													
Subjective quality of life (WHOQOL-BREF)	x						x						x
Motivation for Change Questionnaire (CQ)	x						x						x
**Only intervention group (SEE)**													
Efficacy belief (JSSE-O)			x										
Work alliance (WAI-VR)			x										x
Documentation of the SEE-specialists		x	x	x	x	x	x	x	x	x	x	x	x

## Methods: participants, interventions, and outcomes

### Study setting {9}

The recruiting period started in October 2022 and will end on December 31, 2023. Participants are recruited from outpatient units across the six involved sites:

**Table:** 

***Berlin I***	Vivantes Hospital Am Urban and Vivantes Hospital im Friedrichshain, Charité Universitätsmedizin Berlin, Department of Psychiatry, Psychotherapy and Psychosomatic Medicine, Dieffenbachstraße 1, 10967 Berlin, Germany
***Berlin II***	Charité Universitätsmedizin Berlin with a) Department of Child and Adolescent Psychiatry, Psychosomatic Medicine and Psychotherapy, Campus Virchow Klinikum, Augustenburger Platz 1, 13353 Berlin, Germany, b) Department of Psychiatry and Psychotherapy, Campus Mitte, Charitéplatz 1, 10117 Berlin, Germany, c) Psychiatric University Hospital at St. Hedwig’s Hospital. Große Hamburger Straße 5-11, 10115 Berlin, Germany
***Ulm/Günzburg***	Department of Psychiatry II, Ulm University, District Hospital Günzburg, Lindenallee 2, 89321 Günzburg, Germany
***Munich***	Department of Psychiatry and Psychotherapy, University Hospital LMU Munich, Nussbaumstraße 7, 80338 Munich, Germany
***Hamburg***	Department of Psychiatry and Psychotherapy, University Medical Center Hamburg-Eppendorf, Martinistraße 52, 20246 Hamburg, Germany
***Reichenau***	Zentrum für Psychiatrie Reichenau, Department of Social Psychiatry, Feursteinstraße 55, 78479 Reichenau, Germany

We assume a high representativeness of the SEEearly study sample compared to clinical routine samples, because study settings vary in terms of geographical areas across Germany (north-west: Hamburg, middle east: Berlin I, Berlin II; south: Munich, Ulm/Günzburg, Reichenau), academic (Berlin II, Ulm/Günzburg, Munich, Hamburg) vs. non-academic (Berlin I), and metropolitan (Berlin I, Berlin II, Hamburg, Munich) vs. non-metropolitan areas (Ulm/Günzburg, Reichenau).

### Eligibility criteria {10}

Inclusion criteria are [1] adolescents and young adults aged between 16 and 35 years with [2] a clinical diagnosis of early psychosis. The latter is being defined in accordance with the largest trial of early intervention in psychosis worldwide so far (*n* = 404; [6]): SEEearly participating patients have to fulfill the DSM-5 (Diagnostic and Statistical Manual of Mental Disorders, volume 5) schizophrenia spectrum criteria or other psychotic disorder criteria. In addition, the onset of the first episode of psychosis should not be longer than 5 years ago or initial presentation to mental health services due to psychotic symptoms was within the last 5 years. Structured Clinical Interview for DSM-5 (SCID), Modules B and C, is used to confirm diagnoses according to the DSM-5. Further inclusion criteria are [3] general interest in competitive employment and/or mainstream education and [4] sufficient German language abilities (≥ A2). Exclusion criteria are [1] learning disability or mental retardation, [2] insufficient German language abilities (< A2), and [3] physical or organic handicap that seriously impedes work or educational functioning.

### Who will take informed consent? {26a}

Recruitment is taking place in the outpatient units of the involved sites. Clinicians and/or psychologists refer patients interested in participating in the study to a research assistant (psychologist) who, in turn, schedules an appointment. The research assistant assesses the patients for eligibility, provides written and verbal information about the research project, and answers existing questions before signing the informed consent. Underage participants need the consent of a parent or guardian (Participant Information Sheet for Adults, Adolescents, Custodian and Participant Consent Form in German are available from the corresponding author on request).

### Additional consent provisions for collection and use of participant data and biological specimens {26b}

N/A. There are no additional consent provisions as participant data will only be used for the purpose of the SEEearly trial. Biological specimens are not collected.

## Interventions

### Explanation for the choice of comparators {6b}

In SEEearly, we compare TAU plus SEE (intervention) to TAU alone (control condition) in the respective recruitment centers for 12 months. TAU is defined as common multidisciplinary clinical practice for adolescents and young adults with early psychosis. This includes medical review, pharmacological treatment, psychosocial support including social work counseling, and referral to external government-funded vocational programs. Type and frequency of the psychosocial support interventions in the control condition is being documented. Pharmacological treatment is continuously assessed and the respective chlorpromazine equivalents are calculated. To standardize support regarding work and education across study sites and to make sure that employment and education is being addressed with participants of the TAU condition as well, all social workers involved with participants in the TAU condition are obligated to have at least one consultation with the participants regarding competitive employment and/or mainstream education. Participants of the TAU condition do not have access to SEE treatment during the time of the trial but will be offered IPS after completing the study, if available at the respective mental health service.

### Intervention description {11a}

SEE is based on the nine IPS principles (SE and SEd [Supported Education]). IPS is an evidence-based practice for helping people with severe mental illness to gain and maintain competitive employment and/or mainstream education [16–18, 29]. SEE is delivered by SEE-specialists trained according to a standardized SEE manual [18], which has been translated into German in the preparation phase of the project. Every participant is being assigned to a SEE-specialist, who provides services based on the IPS principles. These include that (a) the SEE-specialist builds partnerships with employees and education program staff and provide follow-up support to both the employee and the employer once work is obtained and (b) the SEE-specialist is part of the mental health treatment team and is therefore located in the same office space and also attends and participates in client’s treatment teams using a shared case management and documentation system.

For the present trial, interventions take place for a duration of 12 months for each participant in the intervention group and range from engagement techniques (e.g., motivational interviewing) to individualized competitive employment and/or mainstream education searches and from experience-based assessment to benefits in counseling/work incentives planning. In cases where participants are hard to reach, SEE-specialists perform repeated attempts to establish contact and document used strategies and the outcome. If contact is not successful over a period of 3 months, the follow-up will stop.

### Criteria for discontinuing or modifying allocated interventions {11b}

There are no known specific risks or side effects of SEE. However, putative side effects of the intervention (e.g., worsening of symptoms, hospitalization, or suicidality) are monitored. An independent data monitoring and safety committee (IDMC) is employed. Participants are able to discontinue the trial at any time without having to give further explanation. Discontinuation has to be documented for the individual participant.

### Strategies to improve adherence to interventions {11c}

The fidelity of SEE is being assessed every 3 months throughout the trial by the IPS Fidelity Scale for Young Adults. This is a 35-item scale developed by the IPS Employment Center [30] and presents a diversification of the IPS-25 fidelity scale [31] specifically tailored to the young adult population. It has two components that are scored separately: the IPS-EMP(loyment) part comprises 25 employment items while the IPS-ED(ucation) part comprises nine education items and one family contact item. Sum Scores range from 0 to 125 for the EMP-part and from 0 to 50 for the Ed-part of the Fidelity Scale. The implementation fidelity refers to the degree to which an intervention is delivered as intended. An IPS-EMP score of 100 or higher indicates good fidelity in IPS employment while an IPS-ED score of 40 or higher indicates good fidelity in IPS education [18, 30].

### Relevant concomitant care permitted or prohibited during the trial {11d}

All participants will receive TAU for adolescents and young adults with early psychosis in an outpatient psychiatric setting for the duration of the trial. Participants of the control group cannot receive SEE for the duration of the trial.

### Provisions for post-trial care {30}

All participants will continue TAU post-trial care. Additionally, participants of the control group will be offered SEE post trial, if available at the respective mental health service.

## Outcomes {12}

### Primary outcome

In reference to international studies [9, 22, 32–34], the primary outcome of SEEearly is the binary indicator “participating steadily over at least 50% of the 12-month follow-up in competitive employment and/or mainstream education,” which includes the dimension of sustained involvement. A similar outcome “participating steadily over at least 50% of the 12-month follow-up in competitive employment” has been frequently proposed as a valid primary outcome to operationalize sustainability of IPS interventions and has been used successfully as primary outcome in IPS RCTs in people with severe mental illness [32, 35].

The primary endpoint was chosen as mainstream education aims to a formal qualification or degree, which includes an extended period of time. The primary outcome will be assessed monthly.

### Secondary outcome

As secondary outcomes, in accordance with relevant studies in the area [36, 37], we monthly assess “*time to first competitive job and/or mainstream education*” (measured in days), “*length of competitive employment and/or mainstream education*” (measured in days), comprising part- and full-time positions, as well as seasonal or temporary positions depending upon the business needs of an employer [18], “*monthly wages*” (measured in Euro) and “*educational attainment*” (measured in degrees/qualifications and ECTS [European Credit Transfer System] points per semester). Furthermore (see also Table 1), secondary non-vocational outcomes are measured at baseline and 6- and 12-month follow-up. It includes psychopathology, functional impairment, substance use, subjective quality of life, and motivation for change. Ongoing psychiatric treatment and ongoing medication as secondary non-employment outcomes are measured monthly.

## Participant timeline {13}

The participant timeline is shown in Fig. 1.

## Sample size {14}

The sample size calculation was based on the primary dichotomous outcome “*steady participation for at least 50% of the 12-month follow-up period in competitive employment and/or mainstream education*” as used in prior studies [22, 32, 38]. Here, the reported steady participation in competitive employment rates vary between 44 and 39% for IPS vs. 11 and 23% for TAU. To detect differences of 24% between the two groups and 40% in the IPS arm vs. 16% in the control arm, the required sample size to find a significant effect with a power of 0.9 at a two-sided significance level of 0.05 is given by 144 participants (72 per arm). The sample size calculation is based on a *χ*^2^-test. As the logistic regression model adjusted for the study site which is used for the primary efficacy analysis yields a power increase compared to the *χ*^2^ test (i.e., the latter ignores the influence of study site effects), this strategy for sample size calculation defines a conservative procedure. The power calculation was performed using NQuery Version 8.4.1.0. Published drop-out and lost to follow-up rates in SE and SEE trials range from 5% [39] to 30% [27]. In a pilot RCT at the study center Berlin I, the drop-out rate was 22%. Assuming a drop-out rate of 22%, the total number of participants to be recruited is thus 184.

## Recruitment {15}

Recruitment is taking place in the outpatient units of the involved sites. Clinicians and/or psychologists refer patients interested in participating in the study to a research assistant who, in turn, schedules an appointment. To secure continuous recruitment of participants, the SEEearly project leader of the sites and the research assistants are in continuous exchange with the clinical staff to provide a constant reminder of the study. Furthermore, study information is available via posters, leaflets in waiting rooms and clinical units and a website.

## Assignment of interventions: allocation

### Sequence generation {16a}

After signing informed consent and immediately after baseline assessment the randomization is carried out, using a computer-generated randomization list that is integrated in the electronic data capture system (EDC). Participants are randomly assigned to one of the two arms with a 1:1 ratio.

### Concealment mechanism {16b}

N/A. Participants are assigned by the SEE-specialists to either intervention or control group immediately after randomization takes place.

### Implementation {16c}

A research assistant (psychologist) assesses the patients for eligibility and provide written and verbal information about the research project and answers existing questions. Before signing the informed consent, underage participants need the consent of a parent or guardian (Participant Information Sheet for Adults, Adolescents, Custodian and Participant Consent Form in German are available from the corresponding author on request). The SEE-specialists perform the randomization and treatment allocation. The latter also inform the participant about the results of the randomization. Participants assigned to the intervention group start the SEEearly treatment at the latest 1 week after allocation. Participants assigned to the control group continue TAU in the outpatient unit.

## Assignment of interventions: blinding

### Who will be blinded {17a}

It is not feasible nor possible for the participating patients and most project staff to be blinded to treatment allocation. However, the research assistants assessing the primary outcome are blind to the intervention group. Participants are instructed not to disclose details of their treatment to the research assistants.

### Procedure for unblinding if needed {17b}

N/A. Research assistants assessing primary and secondary outcomes will not be unblinded during the trial. If unblinding occurs (e.g., the participant reveals allocated intervention to the research assistant), this will be documented in the participant’s file.

## Data collection and management

### Plans for assessment and collection of outcomes {18a}

Comprehensive baseline assessment and 6 and 12 months of follow-up assessments are done face-to-face or, if not possible, via video-calls by a research assistant. Each research assistant has completed a specific training prior to the start of the trial. Monthly assessment of the primary outcome, current medication, and psychiatric treatment is done face-to-face, via video-calls, or telephone. Loss of masking of treatment allocation is being documented. The reliability and validity of assessments will be examined. The baseline assessment includes diagnoses according to ICD-10 (International Statistical Classification of Diseases and Related Health Problems, version 10) and DSM-5, sociodemographic information, including education/employment and housing situation, psychiatric treatment prior to the trial, and motivation for change regarding employment/education. Furthermore, we will use the following validated questionnaires to assess mental health status, substance use, and quality of life at baseline and the follow-ups:

- *Psychopathology* will be measured with the well-established observer-rated Positive and Negative Syndrome Scale (PANSS; [40]). This scale is widely used and known as the “gold standard” for psychopathological outcomes of interventions in people with psychotic disorders [41].
- *Functional impairment* with regard to occupational activity and participation will be assessed due to its tight connection to the aim of SEE. This will be realized by observer ratings on the short version of the International Classification of Functioning, Disability, and Health (Mini-ICF-APP) instrument. This widely applied instrument is used to quantify disability [42] and allows for comparing results of the presented trial with other RCTs [43]. As a global assessment to measure functionality and to ensure comparability with other studies, we also use the Global Assessment of Functioning (GAF; [44]).
- *Substance use* history and past month substance use will be measured by a modified version of the Addiction Severity Index (ASI; [45–47]. Since the comorbidity of *cannabis use* and psychosis has been widely discussed, a German version of the Daily Sessions, Frequency, Age of Onset and Quantity of Cannabis Use Inventory (DFAQ-CU) will be used to specifically assess abuse of cannabis with a focus on frequency, age of onset, and quantity of consumption [48].
- *Subjective quality of life* will be measured with the WHOQOL-BREF [49], a short version of the WHOQOL-100 (World Health Organization Quality of Life scale). This self-rating instrument presents with high rates of internal consistency (*α* = 0.57 to *α* = 0.88) and is translated into 30 languages, which allows for comparison with international research results.
- Economic outcomes will be assessed in terms of *social return on investment* (SROI) at last follow-up after 12 months. This measure is designed to survey the value of social benefits created by a program in relation to the relative cost of achieving those benefits. SROI will be computed as the ratio of “benefits” to “total investment” for each participant and is expressed as percentage. Participants’ earnings in both competitive and non-competitive jobs, education, and apprenticeship hereby account as “benefits.” “Investments” are defined at the total vocational program costs per patient and total costs of mental health service [22, 50–52].

For the intervention group specifically, we included two instruments analyzing *efficacy belief and work alliance*: the Job Search Self-Efficacy scale (JSSE-O), which bases on the tripartite efficacy beliefs model and examines components of self-efficacy, and other efficacies and relation-inferred self-efficacy [53]. The scale consists of 10 items measuring participants’ belief about the success of the job search. Additionally, we included the WAI-VR [54], a modified version of the Working Alliance Inventory (WAI; [55]). It consists of 12 items that examine the factors bond, task and goal.

### Plans to promote participant retention and complete follow-up {18b}

In cases where participants are hard to reach for follow-up, the research assistant performs repeated attempts to establish contact and document used strategies and the outcome. If contact is not successful over a period of three months, the follow-up will stop.

In the event of premature withdrawal from the trial, previously collected data will be included in the evaluation if the participant does not object this procedure.

### Data management {19}

Data is being collected using a paper–pencil based case report form (CRF) during the assessment and is then transferred by the research assistants to an electronic case report form (eCRF) in REDCap run on a secured server provided by the Clinical Trial Office at Charité Berlin Institute of Health (Charité BIH).

Data collection, analysis, and publication will be done using a project-generated identification (ID). The lists linking the IDs with the participants are stored separately from the data. Only the project coordinator and those who have been given access for organizational reasons have access to the link. Quality of data is promoted by range, validity, and consistency checks. Implausible or missing data can be corrected after consultation with the project manager.

### Confidentiality {27}

The study is adhering to the principles of the Helsinki Declaration and monitoring will be performed according to the guidelines on Good Clinical Practice (GCP) by the International Council for Harmonisation of Technical Requirements for Pharmaceuticals for Human Use (ICH). Data collection, analysis, and publication will be done using a project-generated ID. The lists linking the IDs with the participants are stored separately from the data. Only the project coordinator and those having been given access for organizational reasons have access to the link. The study is conducted in accordance to regulatory requirements of the local ethics committees of the involved sites.

### Plans for collection, laboratory evaluation, and storage of biological specimens for genetic or molecular analysis in this trial/future use {33}

N/A. Biological specimens are not collected.

## Statistical methods

### Statistical methods for primary and secondary outcomes {20a}

#### Primary outcome

Confirmatory analysis will be conducted based on the intention-to-treat (ITT) principle. The primary efficacy aim is to show that the percentage of participants who participate steadily over at least 50% of the 12-month follow-up in competitive employment and/or mainstream education is higher in the intervention group than in the control group. A logistic regression model adjusted for study site will be applied with a 95% confidence interval for group comparison. The two-sided significance level is 0.05.

#### Secondary outcomes

As a sensitivity analysis to the primary efficacy, a logistic regression model will be applied with additional covariates age and gender. Descriptive methods will be used for the analyses of the other secondary outcomes, including calculation of appropriate summary measures of the empirical distribution as well as 95% confidence intervals and calculation of descriptive two-sided p-values.

### Safety

Safety analysis includes calculation of frequencies and rates of serious adverse events (SAE). Data will be analyzed using validated statistical software.

### Interim analyses {21b}

N/A. Interim analyses will not be performed.

### Methods for additional analyses (e.g., subgroup analyses) {20b}

Additional sensitivity analyses will be conducted for different populations (per-protocol population, participants with complete cases). Sub-group analyses will be conducted regarding participants’ status of mainstream education at baseline (interrupted mainstream education vs. not engaged in mainstream education).

### Methods in analysis to handle protocol non-adherence and any statistical methods to handle missing data {20c}

Efforts are made to follow-up participants who dropped out from treatment in order to collect outcome data to reduce missing and to detect underlying structures about missing data mechanisms (e.g., missing at random, MAR). Missing values will be imputed using multiple imputation chained equations (MICE; [56]).

### Plans to give access to the full protocol, participant level-data and statistical code {31c}

N/A. We do not plan to grant public access to the full protocol and statistical code. Participant-related data will not be shared at any point of the trial.

## Oversight and monitoring

### Composition of the coordinating center and trial steering committee {5d}

The project leader has regular contact (online or telephone) with project leaders of the involved sites in order to identify potential challenges. The transregional project coordinator supervises the project status and number of study enrollment of each site and holds monthly cross-site online meetings with the assessors and informs the project leader about the current status.

### Composition of the data monitoring committee, its role and reporting structure {21a}

An IDMC will supervise the trial and ensure adherence to the protocol. In case of unexpected, problematic events, they will be asked for advice whether to continue, modify, or stop the trial. It is not planned to conduct interim statistical data analyses while the trial is ongoing.

Further information about names and affiliation of the IDMC are available upon request.

### Adverse event reporting and harms {22}

All study participants are regularly monitored by SEE-specialists, trial staff, and further medical staff. Although there are no known adverse events (AE) or SAE, which have been described as associated with the experimental intervention, an independent expert board is established, which gives advice to the trial staff and monitors safety data. All SAE (e.g., severe worsening of symptoms, hospitalization, or suicidality) reported by the subject or observed by the investigators are documented and assessed to ensure a sufficient surveillance on the safety of the patients. SAE are events that are fatal or life threatening, require hospitalization, or result in persistent or significant disability or incapacity. These events do not necessarily have been caused by the study intervention. Patients must be observed after these events until symptoms have disappeared. SAE are documented in the patient’s chart and on a separate reporting form. They are reported to the coordinating investigator and to the IDMC. A safety surveillance system is established and follows the applicable legislation. Definition on SAE and all other safety information are used as defined by applicable law/guidelines and the protocol. The procedure will follow the standards and standard operating procedures (SOP).

### Frequency and plans for auditing trial conduct {23}

During the trial, quality control is ensured by an on-site monitoring. The Charité-BIH Clinical Trial Office (CTO) coordinates, implements, and conducts the monitoring according to ICH GCP guidelines. This includes on-site initiation, three regular on-site visits, and a closure visit. The on-site review focuses on key data, e.g., signed informed consent, compliance with inclusion/exclusion criteria, and documentation of the primary outcome.

### Plans for communicating important protocol amendments to relevant parties (e.g., trial participants, ethical committees) {25}

Amendments to the study protocol are made by the principal investigator together with the project coordinator. Small amendments are accumulated and then sent to the ethics committee and co-investigators; substantive protocol amendments are sent immediately. The trial registry is updated simultaneously.

### Dissemination plans {31a}

Trial results will be reported according to the applicable CONSORT statement (www.consort-statement.org). The following strategies for the dissemination of the results will be used: publication of trial results in international scientific journals, discussion of trial results during national and international conferences, distribution of trial results by national and international clinical networks as well as through national and international organizations and societies (e.g., Deutsche Gesellschaft für Psychiatrie und Psychotherapie, Psychosomatik und Nervenheilkunde [DGPPN], European College of Neuropsychopharmacology [ECNP], International Early Psychosis Association [IEPA]). Furthermore, study design and progress of the study as well as results of the trial will be presented to people interested in the topic within the scope of relevant peer-lead panels like “*Empower Peers to Research*” (EmPEERie; [57]). Finally, we will inform all study participants about the results of the trial via, for example, an information letter.

## Discussion

There is strong evidence that SE in adults with SMI including young adults with early psychosis results in substantially higher rates of competitive job acquisition, increased working hours per week, and higher wages compared to general rehabilitation services. Based on 30 RCTs [19–21], SE is recommended in national and international treatment guidelines for SMI in general and for schizophrenia in particular [10–12]. Beside vocational outcomes, SE and sustained competitive employment correlate positively with clinical, social, and economical outcomes as well as with quality of life [22–24].

Although interventions in early psychosis are generally regarded as particularly effective in schizophrenia and are highly recommended in the respective guidelines [11, 12], only six RCTs of SE in early psychosis are available up to now [16, 20, 21, 26–28]. In addition, the mentioned trials present with a number of substantial limitations, which prevent SE from being an evidence based strategy in young adults with early psychosis: (a) intervention and follow-up periods of generally only six months are too short to determine the impact of SE on competitive job acquisition [37, 58]; (b) primary outcome is “*at least one day in competitive employment*” rather than “*steady employment or mainstream education*” that is defined as at least 50% of the study period in employment or/and education, although the later has frequently been proposed as more valid primary outcome to operationalize sustainability of the IPS intervention [32, 35]; (c) small sample sizes [58, 59]; (d) a study design that does not allow to determine the relative contribution of SE, because SE was part of a complex intervention [27]; (e) not addressing mainstream education by supplementing SEE [37], although many young adults with first episodes of early psychosis are still in secondary education; (f) not applying fidelity measures [39]; (g) no economic evaluation [21, 27, 58].

The proposed study will be the first multisite RCT of SEE in adolescents and young adults with early psychosis worldwide, which overcomes the limitations of the trials mentioned above. This involves (a) applying SEE rather than SE and (b) considering the primary outcome “*steady participation for at least 50% of the 12 month follow-up period in competitive employment or/and mainstream education*” rather than “*at least one day*,” (c) a sample size that is large enough to determine the relative effects of SEE, (d) applying a meaningful intervention and follow-up time period, (e) measuring the fidelity of the intervention, (f) using a standardized manual of SEE, and (g) applying an economical evaluation. Positive SEEearly results will justify SEE as an evidence-based strategy for clinical routine treatment in early psychosis.

## Trial status

SEEearly was registered in the national and international trial register DRKS (identifier: DRKS00029660) on October 14, 2022. This version refers to version 1.2 of the approved protocol (September 15, 2022). The first participant was enrolled on October 17, 2022. The trial is ongoing and recruiting participants. Recruitment will be completed on 31 December 2023.

## Data Availability

The project leader and the project group will have access to the final dataset. The dataset generated during the current study will not be publicly available, as data sharing was not included in the protocol and consent form on which the ethical approval was based on.

## Abbreviations

AE: Adverse events
ASI: Addiction Severity Index
Charité BIH: Charité Berlin Institute of Health
CRF: Case report form
CTO: Clinical Trial Office
eCRF: Electronic case report form
DFAQ-CU: Daily Sessions, Frequency, Age of Onset, and Quantity of Cannabis Use Inventory
DFG: German Research Foundation
DGPPN: Deutsche Gesellschaft für Psychiatrie und Psychotherapie, Psychosomatik und Nervenheilkunde
DSM-5: Diagnostic and Statistical Manual of Mental Disorders, volume 5
DRKS: German Clinical Trials Register
ECNP: European College of Neuropsychopharmacology
ECTS: European Credit Transfer System
EDC: Electronic data capture system
GAF: Global Assessment of Functioning
GCP: Good Clinical Practice
ICD-10: International Statistical Classification of Diseases and Related Health Problems, version 10
ICH: International Council for Harmonisation of Technical Requirements for Pharmaceuticals for Human Use
ID: Identification
IDMC: Independent data monitoring committee
IEPA: International Early Psychosis Association
IPS: Individual Placement and Support
IPS-ED: IPS-Education
IPS-EMP: IPS-Employment
ITT: Intention-to-treat
JSSE-O: Job Search Self-Efficacy scale
MAR: Missing at random
MICE: Multiple imputation chained equations
OECD: Organization for Economic Cooperation and Development
PANSS: Positive and Negative Syndrome Scale
RCT: Randomized controlled trial
SAE: Serious adverse events
SCID: Structured Clinical Interview for DSM-5
SE: Supported Employment
SEd: Supported Education
SEE: Supported Employment and Education
SMI: Severe mental illness
SOP: Standard operating procedures
SROI: Social return on investment
TAU: Treatment as usual
TVR: Traditional vocational rehabilitation
WAI: Work Alliance Inventory
WHOQOL: World Health Organization Quality of Life scale

## References

[1] Millan MJ, Andrieux A, Bartzokis G, Cadenhead K, Dazzan P, Fusar-Poli P (2016). Altering the course of schizophrenia: progress and perspectives. Nat Rev Drug Discov.

[2] Tandon R, Gaebel W, Barch DM, Bustillo J, Gur RE, Heckers S (2013). Definition and description of schizophrenia in the DSM-5. Schizophr Res.

[3] Jääskeläinen E, Juola P, Hirvonen N, McGrath JJ, Saha S, Isohanni M (2013). A systematic review and meta-analysis of recovery in schizophrenia. Schizophr Bull.

[4] Gore FM, Bloem PJN, Patton GC, Ferguson J, Joseph V, Coffey C (2011). Global burden of disease in young people aged 10–24 years: a systematic analysis. Lancet.

[5] König HH, Friemel S (2006). Gesundheitsökonomie psychischer Krankheiten. Bundesgesundheitsblatt Gesundheitsforschung Gesundheitsschutz.

[6] Kane J, Schooler N, Marcy P, Correll C, Brunette M, Mueser K (2015). The RAISE early treatment program for first-episode psychosis. J Clin Psychiatry.

[7] Bird V, Premkumar P, Kendall T, Whittington C, Mitchell J, Kuipers E (2010). Early intervention services, cognitive-behavioural therapy and family intervention in early psychosis: systematic review. Br J Psychiatry.

[8] Correll CU, Galling B, Pawar A, Krivko A, Bonetto C, Ruggeri M (2018). Comparison of early intervention services vs treatment as usual for early-phase psychosis: a systematic review, meta-analysis, and meta-regression. Effectiveness of early intervention services for early-phase psychosis. JAMA Psychiatry.

[9] Maraj A, Mustafa S, Joober R, Malla A, Shah JL, Iyer SN (2019). Caught in the “NEET trap”: the intersection between vocational inactivity and disengagement from an early intervention service for psychosis. Psychiatr Serv.

[10] DGPPN, editor. S3-Leitlinie Psychosoziale Therapien bei schweren psychischen Erkrankungen. 2nd ed. Berlin, Heidelberg: Springer; 2019.

[11] DGPPN, editor. S3-Behandlungsleitlinie Schizophrenie. Berlin, Heidelberg: Springer; 2019.

[12] NICE. Psychosis and schizophrenia in adults: prevention and management. NICE guidelines [CG178]. 2014. Available from: https://www.nice.org.uk/guidance/cg178. 32207892

[13] Mueser KT, Deavers F, Penn DL, Cassisi JE (2013). Psychosocial treatments for schizophrenia. Annu Rev Clin Psychol.

[14] de Waal A, Dixon LB, Humensky JL (2018). Association of participant preferences on work and school participation after a first episode of psychosis. Early Interv Psychiatry.

[15] Killackey E (2015). Resignation not accepted: employment, education and training in early intervention, past, present and future. Early Interv Psychiatry.

[16] Bond GR, Drake RE, Campbell K (2016). Effectiveness of individual placement and support supported employment for young adults. Early Interv Psychiatry.

[17] Drake RE, Bond GR, Becker DR (2012). Individual placement and support: an evidence-based approach to supported employment.

[18] Swanson SJ, Becker DR, Bond GR, Ellison ML (2020). IPS supported employment for youth: helping transition age youth with serious mental health conditions to access education, jobs, and careers.

[19] Frederick DE, VanderWeele TJ (2019). Supported employment: meta-analysis and review of randomized controlled trials of individual placement and support. PLoS One.

[20] Killackey E, Allott K, Jackson HJ, Scutella R, Tseng YP, Borland J (2019). Individual placement and support for vocational recovery in first-episode psychosis: randomised controlled trial. Br J Psychiatry.

[21] Nuechterlein KH, Subotnik KL, Ventura J, Turner LR, Gitlin MJ, Gretchen-Doorly D (2020). Enhancing return to work or school after a first episode of schizophrenia: the UCLA RCT of Individual Placement and Support and Workplace Fundamentals Module training. Psychol Med.

[22] Hoffmann H, Jäckel D, Glauser S, Mueser KT, Kupper Z (2014). Long-term effectiveness of supported employment: five-year follow-up of a randomized controlled trial. Am J Psychiatry.

[23] Jäckel D, Kupper Z, Glauser S, Mueser KT, Hoffmann H (2017). Effects of sustained competitive employment on psychiatric hospitalizations and quality of life. Psychiatr Serv.

[24] Luciano A, Bond GR, Drake RE (2014). Does employment alter the course and outcome of schizophrenia and other severe mental illnesses? A systematic review of longitudinal research. Schizophr Res.

[25] OECD. Education at a glance 2020: OECD indicators. 2020.

[26] Erickson DH, Roes MM, DiGiacomo A, Burns A. “Individual placement and support” boosts employment for early psychosis clients, even when baseline rates are high. Early Interv Psychiatry. 2020. 10.1111/eip.13005. 10.1111/eip.13005 32578960

[27] Rosenheck R, Mueser KT, Sint K, Lin H, Lynde DW, Glynn SM (2017). Supported employment and education in comprehensive, integrated care for first episode psychosis: effects on work, school, and disability income. Schizophr Res.

[28] Sveinsdottir V, Lie SA, Bond GR, Eriksen HR, Tveito TH, Grasdal AL (2019). Individual placement and support for young adults at risk of early work disability (the SEED trial). A randomized controlled trial. Scand J Work Environ Health.

[29] Bond GR, Drake R, Luciano A (2015). Employment and educational outcomes in early intervention programmes for early psychosis: a systematic review. Epidemiol Psychiatr Sci.

[30] IPS Employment Center. IPS fidelity scale for young adults. IPS Employment Center; 2019. Available from: https://ipsworks.org/wp-content/uploads/2019/03/IPS-fidelity-scale-for-young-adults-3-27-19.pdf.

[31] Becker DR, Swanson S, Bond GR, Merrens MR. Evidence-based Supported Employment fidelity review manual. 2nd edn. 2011. Available from: http://sites.dartmouth.edu/ips/fidelity/fidelity-review-manual/.

[32] Bond GR, Kukla M (2011). Is job tenure brief in Individual Placement and Support (IPS) employment programs?. Psychiatr Serv.

[33] Davis LL, Blansett CM, Mumba MN, MacVicar D, Toscano R, Pilkinton P (2020). The methods and baseline characteristics of a VA randomized controlled study evaluating supported employment provided in primary care patient aligned care teams. BMC Med Res Methodol.

[34] Rogers ES, Kash-MacDonald M, Bruker D, Maru M (2010). Systematic review of supported education literature 1989–2009.

[35] Bond GR, Campbell K, Drake RE (2012). Standardizing measures in four domains of employment outcomes for Individual Placement and Support. Psychiatr Serv.

[36] HegelstadWenche ten V, Joa I, Heitmann L, Johannessen Jan O, Langeveld J (2019). Job- and schoolprescription: a local adaptation to individual placement and support for first episode psychosis. Early Interv Psychiatry.

[37] Killackey E, Allott K, Cotton SM, Jackson H, Scutella R, Tseng Y-P (2013). A randomized controlled trial of vocational intervention for young people with first-episode psychosis: method. Early Interv Psychiatry.

[38] Davis LL, Kyriakides TC, Suris AM, Ottomanelli LA, Mueller L, Parker PE (2018). Effect of evidence-based Supported Employment vs transitional work on achieving steady work among veterans with posttraumatic stress disorder: a randomized clinical trial. JAMA Psychiatry.

[39] Killackey E, Allott K, Woodhead G, Connor S, Dragon S, Ring J. Individual placement and support, supported education in young people with mental illness: an exploratory feasibility study. Early Interv Psychiatry. 2017:Advance online publication. 28.04.2016. 10.1111/eip.12344. 10.1111/eip.12344 27121481

[40] Kay SR, Fiszbein A, Opler LA (1987). The positive and negative syndrome scale (PANSS) for schizophrenia. Schizophr Bull.

[41] Opler MGA, Yavorsky C, Daniel DG (2017). Positive and Negative Syndrome Scale (PANSS) Training: challenges, solutions, and future directions. Innov Clin Neurosci.

[42] Linden M, Baron S, Muschalla B (2010). Relationship between work-related attitudes, performance and capacities according to the ICF in patients with mental disorders. Psychopathology.

[43] Rössler W, Ujeyl M, Kawohl W, Nordt C, Lasalvia A, Haker H (2019). Predictors of employment for people with mental illness: results of a multicenter randomized trial on the effectiveness of placement budgets for Supported Employment. Front Psychiatry.

[44] Aas IHM (2010). Global Assessment of Functioning (GAF): properties and frontier of current knowledge. Ann Gen Psychiatry.

[45] McLellan AT, Carise D, Coyne TH (1992). Addiction Severity Index 5th Edition. J Subst Abuse Treat.

[46] Leonhard C, Mulvey K, Gastfriend DR, Shwartz M (2000). The Addiction Severity Index: a field study of internal consistency and validity. J Subst Abuse Treat.

[47] Rosen CS, Henson BR, Finney JW, Moos RH (2000). Consistency of self-administered and interview-based Addiction Severity Index composite scores. Addiction.

[48] Cuttler C, Spradlin A (2017). Measuring cannabis consumption: psychometric properties of the Daily Sessions, Frequency, Age of Onset, and Quantity of Cannabis Use Inventory (DFAQ-CU). PLoS One.

[49] Angermeyer MC, Kilian R, Matschinger H (2000). WHOQOL-100 und WHOQOL-BREF. Handbuch für die deutsche Version der WHO Instrumente zur Erfassung von Lebensqualität.

[50] Millar R, Hall K (2013). Social Return on Investment (SROI) and Performance Measurement: the opportunities and barriers for social enterprises in health and social care. Public Manag Rev.

[51] Phillips JJ (2003). Return on investment in training and performance improvement programs.

[52] Luppa M, Luck T, Heinrich S, Glaesmer H (2008). Forschung zur Versorgung von Patienten mit psychischen Störungen. Z Psychiatr Psychol Psychother.

[53] Jackson B, Dimmock JA, Taylor IM, Hagger MS (2012). The tripartite efficacy framework in client-therapist rehabilitation interactions: implications for relationship quality and client engagement. Rehabil Psychol.

[54] Chan F, McMahon BT, Shaw LR, Lee G (2004). Psychometric validation of the expectations about rehabilitation counseling scale: a preliminary study. J Vocat Rehabil.

[55] Horvath AO, Greenberg LS (1989). Development and validation of the Working Alliance Inventory. J Couns Psychol.

[56] van Buuren S, Groothuis-Oudshoorn K. mice: Multivariate imputation by chained equations in R. J Stat Softw. 2011;45(3):1–67.

[57] Demke E, Mahlke C, Bock T (2017). EmPeeRie-Empower Peers to Research-Vorstellung eines Hamburger Projekts zur Förderung von Partizipativer und betroffenenkontrollierter Forschung. Sozialpsychiatrische Informationen.

[58] Killackey E, Jackson HJ, McGorry PD (2008). Vocational intervention in first-episode psychosis: individual placement and support v. treatment as usual. Br J Psychiatry.

[59] Humensky JL, Turner LR, Dixon LB, Drake RE, Becker DR, Subotnik KL (2020). Personnel time required for supported employment and education services for individuals in a recent-onset psychosis treatment program. Early Interv Psychiatry.

