# Correction: Enhancing educational and vocational recovery in adolescents and young adults with early psychosis through Supported Employment and Education (SEEearly): study protocol for a multicenter randomized controlled trial

**DOI:** 10.1186/s13063-023-07524-5

**Published:** 2023-07-25

**Authors:** D. Jäckel, A. Willert, A. Brose, K. Leopold, D. Nischk, S. Senner, O. Pogarell, S. Sachenbacher, M. Lambert, A. Rohenkohl, P. Kling-Lourenco, N. Rüsch, F. Bermpohl, M. Schouler-Ocak, V. Disselhof, U. Skorupa, A. Bechdolf

**Affiliations:** 1 Department of Psychiatry and Psychotherapy, Charité Campus Mitte, Charité UniversitätsmedizinBerlin, Berlin, Germany; 2 Department of Psychiatry, Psychotherapy and Psychosomatics, Vivantes Klinikum Am Urban and Vivantes Klinikum Im Friedrichshain, Berlin, Germany; 3 Department of Psychiatry and Psychotherapy, University Hospital Carl Gustav Carus, Technical University Dresden, Dresden, Germany; 4 Department of Social Psychiatry, Zentrum Für Psychiatrie, Reichenau, Germany; 5 Department of Psychiatry and Psychother- Apy, University Hospital, LMU Munich, Munich, Germany; 6 Department of Psy- Chiatry and Psychotherapy, University Medical Center Hamburg-Eppendorf, Hamburg, Germany; 7 Department of Psychiatry II, University of Ulm and BKH Günzburg, Ulm, Germany; 8 Psychiatric University Clinic of Charité at St. Hedwig Hospital, Berlin, Germany

**Correction: BMC Trials 24, 440 (2023)**

**https://doi.org/10.1186/s13063-023-07462-2**

Following publication of the original article [1], we have been informed that Fig. 1 was incomplete.

Originally published Fig. 1:
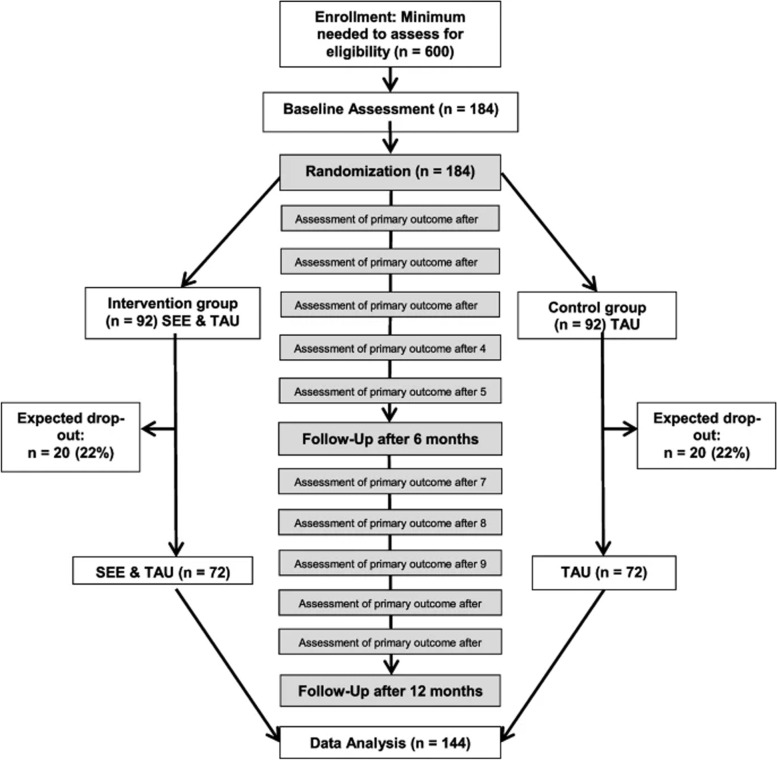


Corrected Fig. 1:
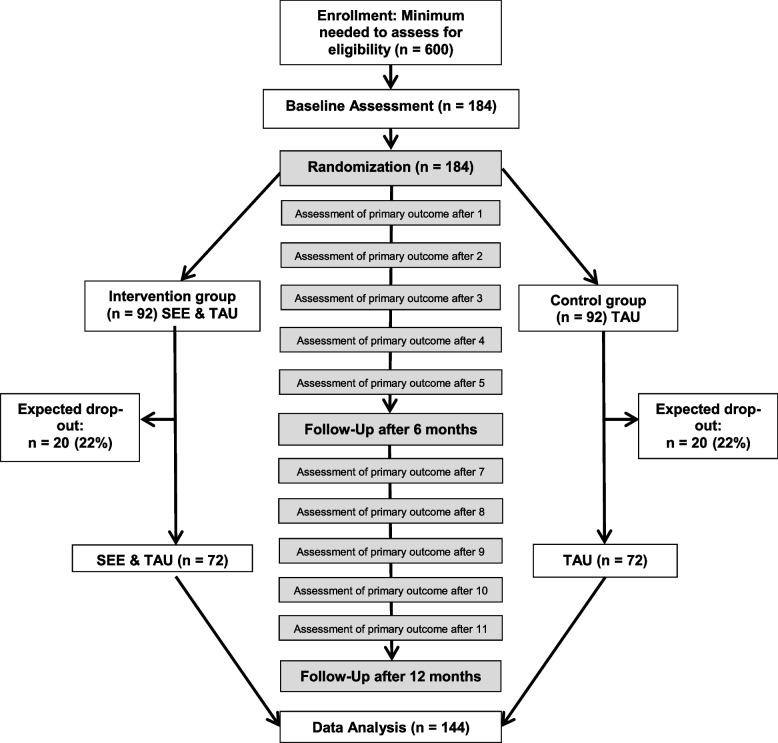


The original article has been corrected.
